# Two strategies of anticipatory mechanism during change in frequency of cyclic multi-finger force production in isometric conditions

**DOI:** 10.1007/s00221-026-07318-6

**Published:** 2026-05-19

**Authors:** Jaeho Park, Hyun-Soo Kim, Yoon-Seok Choi, Sungjune Lee, Narae Shin, Jaebum Park

**Affiliations:** 1 Institute of Sport Science, Seoul National University, #414, 71-1, 1 Gwanak-Ro, Gwanak-Gu, Seoul, 08826 South Korea; 2 Department of Physical Education, Seoul National University, Seoul, South Korea; 3 Advanced Institute of Convergence Technology, Seoul National University, Suwon, South Korea; 4 Department of Kinesiology, Michigan State University, East Lansing, MI USA

**Keywords:** Feedforward mechanism, Motor abundance, Uncontrolled manifold hypothesis, Multi-finger synergy, Anticipatory synergy adjustment

## Abstract

Cyclic force production by multiple fingers requires proper organization of finger forces, i.e., synergy, while concurrently accommodating predictable or anticipatory changes in movement frequency. A plethora of studies have been conducted on this subject, employing an experimental paradigm of the quick pulse force production subsequent to the steady-state condition, while the underlying mechanism of reorganization of multi-finger synergies remains unclear in response to required cyclic dynamics changes. In the present study, the time-varying multi-finger synergy and its anticipatory adjustment were investigated in healthy young adults who generated cyclic four-finger flexion at 0.5 and 2 Hz under “*frequency-maintained*” and “*frequency-changed*” conditions. The participants were informed of the transition in advance. Synergy indices in time-series were quantified across multiple trials using the uncontrolled manifold framework. The synergy indices demonstrated transient decreases exclusively during the “*frequency-changed*” frequency conditions, indicating a temporary weakening of force-stabilizing coordination around the frequency transition. The decrease manifested earlier and more distinctly when frequency changed from high-to-low, whereas in the low-to-high transition, it was delayed by about 30 ms. The observed synergy drop was driven primarily by increased variance orthogonal to the uncontrolled manifold, with relatively little change in variance within the uncontrolled manifold. These findings suggest that predictable cyclic force transitions are accommodated through temporally specific reorganization of multi-finger coordination, expressed mainly as a transient increase in task-relevant variance.

## Introduction

The human body system has the capacity to anticipate and prepare for impending tasks (Bouisset and Zattara 1987; Latash et al. 1995). The collective operation of these mechanisms is described by the term “feedforward mechanism”. Examples of this mechanism can be observed in hand-held object manipulation (Shim et al. 2006), postural adjustment (Aruin and Latash 1995; Horak 2006), the bimanual coordination dynamics approach (Scholz and Kelso 1990), and the internal model approach on the predictive grip (Flanagan and Wing 1997). The measured variables employed for confirming the anticipatory strategies in humans included mechanical variables, such as end-effort forces and center of pressure (COP) (Aruin and Latash 1995; Klous et al. 2011; Park et al. 2012; Riach et al. 1992; Shim et al. 2005), among others, as well as physiological variables, including electromyography (EMG) (Bouisset and Zattara 1981) and more direct measures of the brain activity (Chiou et al. 2018). In contrast, the feedback mechanism in the human motor system pertains to the sensory-based updating of motor commands using the available information about the current state (Scott 2004; Todorov and Jordan 2002). In essence, the feedforward and feedback mechanisms are of neural origin with regard to the neurophysiological “***process***” of the control “***system***”, that is, the central nervous system (CNS). Therefore, the measurement of the physiological variables would be a rational approach for examining various features of the feedforward and feedback mechanisms. However, a considerable proportion of recent studies have utilized a combination of mechanical outcomes and computational frameworks to estimate indices related to neural processes because the mechanical outcomes are the functional consequence or task-level output of the control process. In other words, the stabilization or alternation in the mechanical outcome may be the objective of the controller’s activity (Scott 2004). In this regard, the operational definitions of the aforementioned studies, including the present study where the direct measure of physiological variable was not included are as follows: the feedforward mechanism refers to the anticipatory setting of task-level control variables, whereas feedback refers more broadly to stabilizing processes supported by sensory signals within the control hierarchy.

The feedforward mechanism, a primary keyword of the present study, can be interpreted in a combined manner with the concepts of motor abundance. This interpretation is supported by the experimental findings that demonstrated the beneficial effects of the additional degrees of freedom inherent in the human body system (Kim et al. 2018; Olafsdottir et al. 2005, 2007b; Shim et al. 2005). However, this does not imply that the motor abundance is a stringent prerequisite for comprehending the feedforward mechanism. Rather, the motor abundance system supplies the task-equivalent solution families that facilitate the utilization of the control process in a predictive manner. In other words, the human body system possesses the capacity to modify the combination pattern of involved elements in the absence of apparent changes in overall output for the recognized upcoming tasks. The concept of “***synergy***” has been introduced to depict the proper covariation between the abundant set of elements necessary to satisfy the given motor task. The covariation patterns appear to be partially influenced by the mechanical and physiological characteristics of the task, with the aim of ensuring the stabilization of the net outcome as performance variables. The performance variables are predetermined and stabilized by the voluntary structuring of elements, i.e., synergic organization, ensuring adherence to the mechanics of task constraints (Latash et al. 2001; Scholz and Schöner 1999; Song et al. 2021).

The feedforward mechanism is regarded as the modulation of the covariation pattern between the elements from the perspective of synergy. The modulation constitutes a pivotal function in actual movements, enabling us to attribute to the anticipation and make voluntary changes in body dynamics. The modulation process is partly responsible for the intentional alterations in the magnitude of synergy. Generally, a decrease in synergy magnitude is indicative of a state of destabilization. The term “*system destabilization*” does not exclusively denote adverse alterations in this context. In order to induce a change in the dynamics of the human system by means of a new motor command, such as a change in walking pattern (Ivanenko et al. 2005), changes in hand trajectories (Flanagan et al. 1993), or force (Latash et al. 2002), it is necessary to destabilize the system of the actively involved body elements at a certain phase. Consequently, maintaining stability (i.e., synergy) during the abrupt alterations in net mechanical outcome (i.e., salient performance variable) is not advantageous. Conversely, deactivating the synergy would be a superior strategy, as the specific covariation pattern for stabilizing a particular performance variable is not of significant importance. The phenomenon of the purposeful destabilization has been observed in 200–400 ms prior to the visible changes in the net outcome, which has been termed the anticipatory synergy adjustment (ASA) (Jo et al. 2016; Olafsdottir et al. 2005; Park et al. 2012; Piscitelli et al. 2017; Shim et al. 2005; Zhou et al. 2013).

Our current curiosity was piqued, and we were compelled to explore the strategy that anticipated or prepared a change in covariation patterns of the individual finger forces when the time frame was sufficient and not sufficient, along with the net force that was supposed to change during the anticipation or preparation phase. Consequently, the present experiment employed the multiple cyclic force production paradigm. The task of the present paradigm was hypothesized to be more challenging and divergent from the paradigm of the preceding studies, i.e., steady-state force production since the cyclic force production necessitates scaling of the net outcome continuously. The mathematical model of motor variability, also known as the *linear model*, was proposed by Gutman (Gutman et al. 1993; Gutman and Gottlieb 1992). This model posits that the cyclic force production naturally demands the changes in the variance observed in the orthogonal complement to the subspace where various combinations of redundant elements do not change the net outcome, i.e., the null space. In accordance with the prevailing terminologies employed in the uncontrolled manifold (UCM) computation, the observed variance in the orthogonal subspace with respect to the null space has been designated as “error variance”, which depicts the variability in the task-relevant direction related to the chosen performance variable. Consequently, the alternation in frequency of cyclic force scales the rate of force changes (*dF*/*dt*), and this should be associated with the changes in the error variance, which causes the changes in the synergy indices as the stability indicator. A notable challenge in the change in cyclic force production may be the transition of the frequency of cyclic force from high to low. For instance, the anticipatory adjustment was set to a duration of 250 ms at a frequency of 2 Hz. Therefore, it is conceivable that this temporal span may present a significant challenge in modulating the variance components. Conversely, the frequency undergoes a transition from low to high, and the temporal window is sufficient (e.g., 1 s for a 0.5 Hz condition) to activate the feedforward mechanism. Furthermore, it is evident that these alternations would be predominantly attributed to the active modulation of the space domain wherein the net force undergoes a change, that is to say, orthogonal space related to the null space of the Jacobian of the prevailing motor task, in conjunction with the time derivative of the net force (*dF*/*dt*).

The objective of the present study is twofold: First, it is imperative to possess a quantitative understanding of the anticipation process at the level of the end-effector force, i.e., finger force during cyclic tasks, wherein the net finger force is kept changed. Secondly, the study investigated the frequency dependency of the synergy formation and its modulation with the feedforward mechanism by observing the modulation of the synergy and along with sub-variance components during cyclic force production. On the basis of prior knowledge and experimental findings on previous experimental outcomes about the feedforward changes (Jo et al. 2016; Olafsdottir et al. 2005; Park et al. 2012; Piscitelli et al. 2017; Shim et al. 2005), the following hypotheses were formulated: 1) The anticipatory synergy adjustment (ASA) would be observed (i.e., the drop in the synergy index) even during cyclic force production, as the pulse force production task demonstrated. In particular, it is expected that the synergy-drop would be more evident when the required cyclic frequency is supposed to change from the low to high conditions rather than from the high to low change. This is due to the time limitation for the changes in the covariation pattern between the elements, that is, the individual finger forces. 2) The synergy index exhibits a decline in the anticipatory manner. That is to say, prior to the frequency change, the variance along the task-relevant direction (i.e., error variance observed in the orthogonal space to the null space) would play a significant role in the reduction of the synergy index. 3) The application of the linear model would result in a diminished relationship between the rate of force changes and the error-variance during the time window of anticipatory synergy adjustment. This phenomenon can be attributed to the potential for a reduction in force variability, which, in turn, may lead to an increased likelihood of destabilization.

## Methods

### Participants

The study was conducted on a group of twelve healthy young adults (age: 25.2 ± 1.9 yrs; 174.3 ± 6.3 cm; 69.4 ± 6.5 kg). All participants were right-handed, as determined by the Edinburgh Handedness Inventory (Oldfield 1971). It is noteworthy that none of the participants had a medical history of neurological or musculoskeletal disorders, nor had they sustained injuries to their upper limbs. The Institutional Review Board (IRB) at Seoul National University formally endorsed the experimental protocol (IRB No. 2503/003-003). The participants provided their signatures on the approved consent form, thereby affirming their voluntary participation, their awareness of the experimental procedures, and their right to withdraw from the experiment at any time during its course.

### Experimental setup and apparatus

**Fig. 1:**
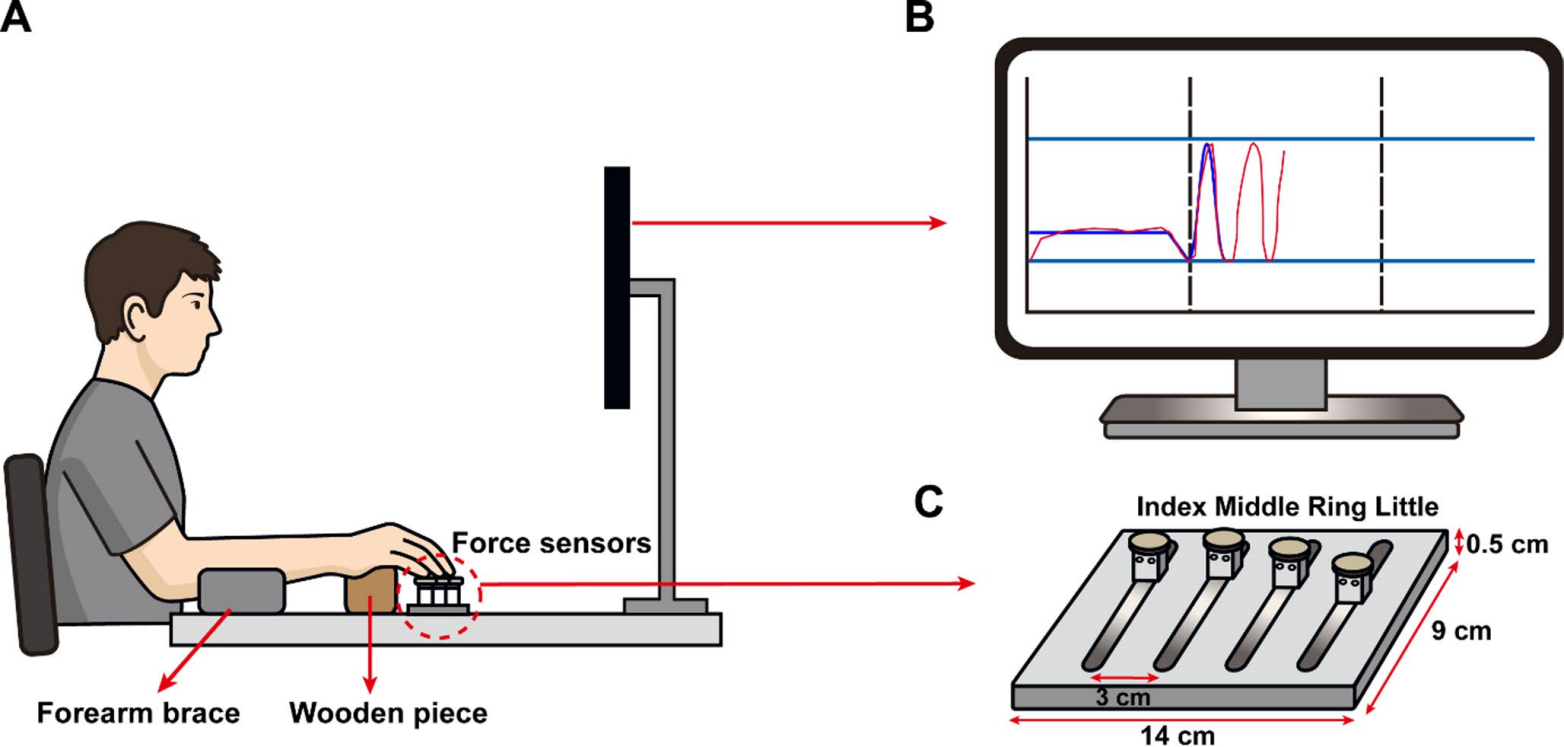
**A** Experimental setup. Participants sat in front of a monitor and produced force with the right hand by pressing force sensors while the forearm brace and wooden piece were adjusted to a comfortable position. **B** Visual feedback displayed on the monitor. Two horizontal lines indicated the target force peak and trough, and two vertical dashed lines indicated the onset of cyclic force production and the timing of the frequency transition. A steady-state force guide lasting 10 s and the first-cycle force template (blue trace) were also provided. The total force produced by the four fingers was displayed in real time as a red trace. **C** Customized finger task frame. Four single-axis force sensors were positioned to align with the index, middle, ring, and little fingers

Four single-axis force sensors (SSBA-S, Curiotec Co. Ltd, Korea; Fig. 1C) were used to measure individual finger flexion forces in an isometric condition. Each sensor was meticulously abraded with 200-grit sandpaper to ensure adequate friction and to avert the occurrence of slippage on the surface of the fingertips. Four sensors were mounted on a customized experimental frame (0.14 m × 0.09 m × 0.005 m, Fig. 1C) at a distance of 0.03 m between the two adjacent force sensors in the medio-lateral direction. Finger force data were digitally sampled at 100 Hz, and the data were collected using an analog-to-digital converter that was connected to a customized LabVIEW program (National Instruments, Austin, TX, USA). The height of the chair and monitor was adjusted to ensure comfort for the participants. The wooden piece and forearm brace were positioned in a manner that constrained the elbow posture. The right shoulder was maintained at approximately 45° of abduction, and 30° of flexion, and the elbow was flexed about 60° (Fig. 1A). In order to mitigate potential distractions and facilitate sustained concentration, blinds were installed to cover participants’ visual field, except for the front view.

### Experimental procedures

The tasks were comprised of two components. The initial component constituted an auxiliary session that measured the maximal voluntary contraction of the net finger, i.e., all four fingers, flexion force (MVC_TOT_). The subsequent session pertained to the primary task, which entailed the execution of cyclic flexion force production tasks under various frequency conditions. Participants were instructed to perform the designated tasks with their right hand in an isometric condition. The net flexion forces exerted by the four fingers were measured and displayed in real time on a computer screen to the participants. The computer screen measured 27 inches in diagonal length, and its refresh rate was configured at 120 Hz.

#### Maximal voluntary contraction (MVC) finger force production task

The participants were instructed to apply a flexion force by pressing with all four fingers, gradually increasing the net force until it reached its maximal level. Two trials were conducted, with each trial lasting 10 s. A 3 min rest period was implemented between trials to minimize fatigue. The mean of the peak force values from the two trials was defined as the participant's maximum voluntary contraction force (MVC_TOT_). This MVC_TOT_ was utilized to ascertain the target force values for the next task, the cyclic force production task.

#### Cyclic net finger force production task divided into two frequency conditions in terms of time sequence

**Fig. 2:**
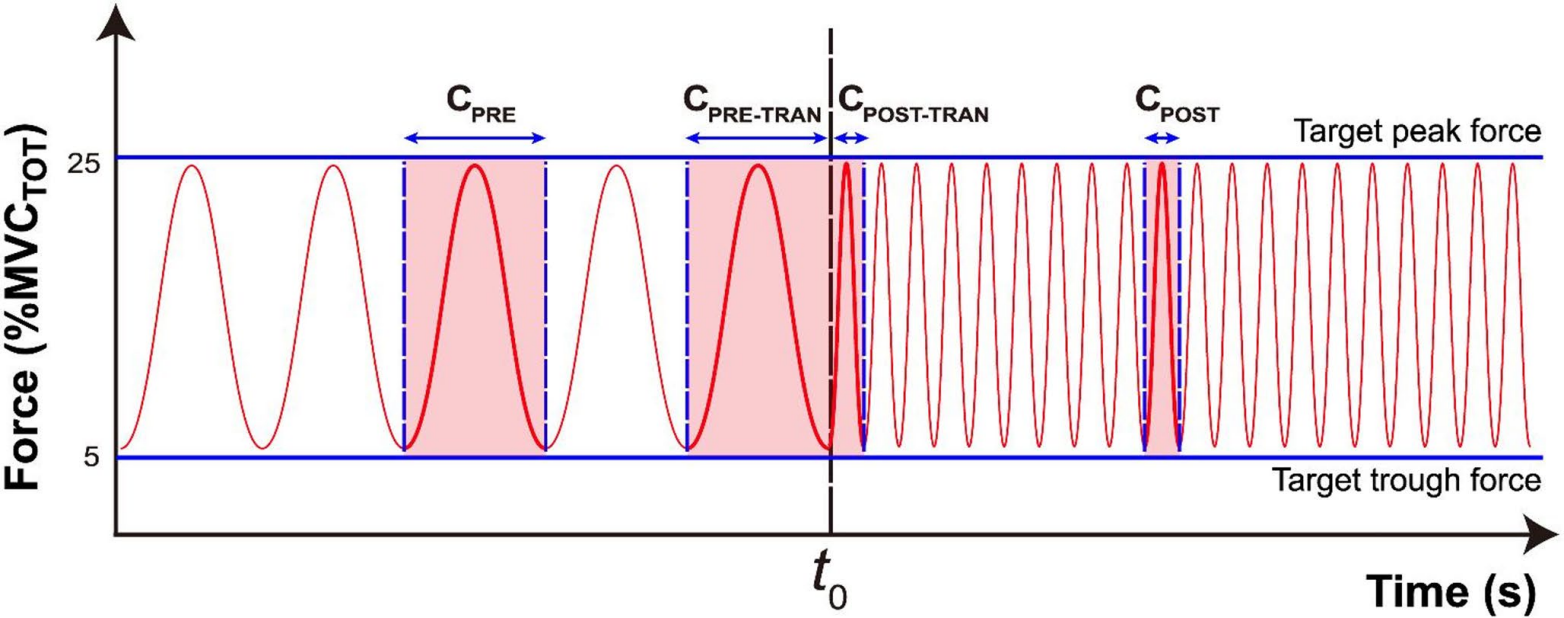
Segmentation of cyclic force production. For analysis, four distinct single cycles were selected from the cycles produced during a 20 s trial. The HL condition (transition from 2 to 0.5 Hz) is shown as an example. One cycle randomly selected from the 10 s period before the frequency transition was defined as C_PRE_. The cycle just before the frequency transition point *t*_0_ was defined as C_PRE-TRAN_. The cycle immediately following the frequency transition was defined as C_POST-TRAN_, and one cycle randomly selected from the 10 s period following the frequency transition was defined as C_POST_

The primary task of the present study was to assess the performance of the cyclic flexion force production task utilizing all four fingers. During the trial, the total finger force (F_TOT_) was displayed in real-time on the computer screen. The frequencies of the cyclic force were altered or maintained with respect to the midpoint of the single trial (Fig. 2). Therefore, the conditions were comprised of two distinct categories, with each category containing two sub-conditions, resulting in a total of four conditions. The four conditions of the frequency modulation comprised of “***changed***”, 1) 0.5 Hz to 2 Hz (low to high, **LH**) and 2 Hz to 0.5 Hz (high to low, **HL**) and “***maintained***”, 3) 0.5 Hz to 0.5 Hz (low to low, **LL**) and 4) 2 Hz to 2 Hz (high to high, **HH**) of cyclic force profile. In summary, alternation in the frequencies of cyclic force was required in the **LH** and **HL** conditions at the constrained time moment. Each trial lasted 30 s and consisted of three phases of 10 s each (Fig. 1B). The first phase involved 10% of MVC_TOT_ steady-state force production, while the second and third phases focused on the initial frequency and the terminal frequency of cyclic force production, respectively. It is noteworthy that the initial 10 s of steady-state force production were excluded from the data analysis, as this period was allocated for the pre-activation of muscles prior to the subsequent 20 s cyclic force production task. To enhance the participant's recognition of the accuracy of the produced force, a set of reference lines was presented on the computer screen, delineating two vertical and two horizontal directions. The time profile of cyclic force production was delineated by two horizontal lines, indicating the levels of trough (5% of MVC_TOT_) and peak forces (25% of MVC_TOT_) (Latash et al. 2002; Park et al. 2012). The cyclic force production phase was initiated by passing the force cursor to the first vertical line, followed by the steady-state force production. The second vertical line indicated a specific moment in time at which an alternation or maintenance of the frequency of cyclic force production would occur. It is important to note that, except for the initial cycle, the cyclic force template was not provided; only the net finger force was shown on the screen during cyclic force production (Fig. 1B). In lieu of the visual force template, a metronome sound was provided to the participants at both peak and trough to ensure precise timing of cyclic force production.

The overarching instruction to participants entailed the maintenance of a steady-state force, subsequently succeeded by oscillatory force production in a sinusoidal pattern between 5 and 25% flexion force of MVC_TOT_ as accurately as possible. Specifically, the participants were made aware of the conditions of the initial and terminal frequency of the cyclic force production prior to the commencement of the trial. Consequently, a specific instruction was provided to the participants, which read, “*Do not make a significant change in the frequency of force profile before the cursor crosses the second vertical line*,” with particular emphasis on the LH (low 0.5 Hz to high 2 Hz) and HL (high 2 Hz to low 0.5 Hz) conditions. Prior to each condition, the participants were allotted 4–5 min to acclimate to the task. An additional few minutes were designated for the practice of the LH and HL conditions. Each participant completed 20 trials for each of four conditions, LH, HL, LL, and HH, and the four conditions were block-randomized across all the participants. The entire duration of the experiment was approximately 90 min. The duration of the trials and conditions was sufficient to ensure that all participants remained within the acceptable range of fatigue levels throughout the experiment.

### Data analysis

The force data were subsequently filtered with a zero-lag, fourth-order Butterworth filter set at a cutoff frequency of 10 Hz. All analyses were conducted using customized MATLAB codes (MathWorks, Natick, MA, USA). It is imperative to acknowledge that the initial 10 s period of the steady-state force production phase was excluded from further analysis, consequently leading to the utilization of the subsequent 20 s data set for the main analysis. The objective of the present experiment was to generate cyclic force production, wherein the combination of force increase (up cycle, C_UP_) and decrease (down cycle, C_DW_) was imperative. The C_UP_ and C_DW_ were separated or combined, depending on the objectives of the data analysis. A total of twelve participants were recruited for the experiment. Data from two participants were excluded because they withdrew from the study due to scheduling conflicts before completing the full experimental protocol. The final analysis was therefore performed on ten participants.

#### Curation of multiple cycles from multiple trials

In the initial step of the data analysis, four distinct single “***cycles***” were selected from a 20 s single trial, as depicted in Fig. 2. A cycle was defined using a trough-to-trough criterion, that is, from one local minimum (trough) to the subsequent local minimum. Within each cycle, C_UP_ corresponded to the interval from the initial trough to the local maximum (peak), and C_DW_ corresponded to the interval from the peak to the subsequent trough. The acceptance criteria for successful cycles were as follows: the error of either peak or trough force was not to exceed ± 4% of MVC_TOT_ in its magnitude and ± 4% of the time duration of the target frequency condition in its time (0.48–0.52 s for the 0.5 Hz and 0.12–0.13 s for the 2 Hz). Secondly, the selection of a set of four cycles within a single trial was determined as follows: 1) one successful cycle randomly selected from the initial 10 s (C_PRE_), 2) a cycle immediately preceding the initiation of the second frequency performance (C_PRE-TRAN_), 3) the first cycle of the transition to the second frequency performance (C_POST-TRAN_), and 4) one successful cycle randomly selected from the last 10 s (C_POST_). This selective strategy was implemented for all 20 trials of all four conditions, namely LH, HL, LL, and HH. It is noteworthy that the transition cycles, C_PRE-TRAN_ and C_POST-TRAN,_ were exclusive to a single case within a single trial, while the C_PRE_ and C_POST_ were selected randomly from among the successful cycles satisfying the acceptance criteria. The mean number of selected cycles for further analysis was 17.7 ± 1.1 cycles out of 20 trials for both C_PRE-TRAN_ and C_POST-TRAN_, whereas 20 cycles were consistently obtained for C_PRE_ and C_POST_. Across conditions, the mean number of accepted cycles was 19.5 ± 0.5, 19.0 ± 0.6, 16.9 ± 1.6, and 19.8 ± 0.5 cycles out of 20 trials for the LH, HL, LL, and HH conditions, respectively.

#### Performance indices during cyclic finger force production

The performance indices were estimated from the multiple-cycle data from the selected cycles by the aforementioned criteria. Consequently, the number of cycles varied across participants and conditions. To account for this variability, the variables were averaged across accepted cycles within each participant for each condition and cycle prior to statistical analysis. The absolute force error (F_ER_) and mean power frequency (MPF) were identified as the performance accuracy indices, with the estimation of these indices serving as the basis for further analysis. Absolute force error (F_ER_) was estimated at the peak and trough of each cycle, where the trough corresponds to the minimum value following the peak. The combined data from the up (C_UP_) and down (C_DW_) phases were used to compute the MPF. The accuracy of force magnitude was quantified by the absolute error at peak and trough points with respect to the target force level (F_target_) given by the horizontal visual template on the computer screen. This error was then normalized by the MVC_TOT_ of individual participants. The absolute error was then computed as follows (Eq. 1).

1 $${F}_{ER, i}^{j,k}=\frac{1}{N}\sum_{ i=1}^{N}\left|\frac{{F}_{i}^{j,k}-{F}_{target}^{i,j,k}}{{MVC}_{TOT}}\right|\times 100$$

where *i* = {peak, trough}, *j* = {LH, HL, LL, and HH}, and *k* = {C_PRE_, C_PRE-TRAN_, C_POST-TRAN_, and C_POST_}. Additionally, *N* signifies the total number of accepted cycles from all the trials that met the aforementioned criteria, for each participant and four experimental conditions, i.e., LH, HL, LL, and HH.

To assess the performance accuracy for each given frequency condition during the cyclic force production, a fast Fourier transform (FFT) was conducted. In a single trial, the four cycles were selected as described above: C_PRE_, C_PRE-TRAN_, C_POST-TRAN_, and C_POST_. The mean power frequency (MPF) for each participant was first calculated for each of four cycles in a single trial under each frequency condition. Subsequently, the averaged MPF value across trials for each frequency and cycle was obtained using Eq. 2.

2 $${MPF}_{j}^{k}=\frac{{\sum}_{h}{f}_{h}^{j,k}\cdot {P}_{h}^{j,k}}{{\sum}_{h}{P}_{h}^{j,k}}$$

where *f*_*h*_ is the frequency of bin *h* (Hz), and *P*_*h*_ is its power (i.e., power spectral density) value. *j* = {LH, HL, LL, and HH} and *k* = {C_PRE_, C_PRE-TRAN_, C_POST-TRAN_, and C_POST_}.

#### Index of four finger coordination during cyclic force production (for details see Appendix 1)

The objective of this analysis was to quantify the indices of four-finger coordination, synergy (Latash et al. 2002; Scholz and Schöner 1999), during cyclic force production. To this end, the uncontrolled manifold (UCM) computation was performed. The preliminary outcomes of the UCM computation were two components of variance across multiple trials in time series, which were identified in two distinct subspaces: the UCM and the orthogonal complement of the UCM (ORT space). These subspaces were estimated to be spanned by the eigenvectors of the null space and the singular vectors of the corresponding Jacobian. The variance projected data onto the UCM space was referred to as V_UCM_, whereas that onto the ORT space was designated as V_ORT_. Further, the synergy index, namely as ΔV in the time-series of a half sine-wave for a particular frequency condition was computed. This computation was performed separately on the four cycles (C_PRE_, C_PRE-TRAN_, C_POST-TRAN_, and C_POST_) within each of the four frequency modulation conditions. Since the time duration of multiple cyclic forces was slightly different across the trials, the force data for each of the four cycles were resampled to 200 data points using cubic spline interpolation (Friedman et al. 2009; Park et al. 2012), including 100 for the up-cycle (C_UP_) and 100 for the down-cycle (C_DW_).

At each time point, two components of variance were computed. V_UCM_ represented the component of variance that did not affect the task performance variable (i.e., total force), while V_ORT_ reflected the component of variance that led to changes in the total force. The synergy index (ΔV), defined as the relative difference between V_UCM_ and V_ORT_, was then computed as Eq. 3:

3 $$\Delta V\left(t\right)=\frac{{V}_{\mathrm{U}\mathrm{C}\mathrm{M}}\left(t\right)/\left(n-p\right)-{V}_{\mathrm{O}\mathrm{R}\mathrm{T}}\left(t\right)/p}{{V}_{\mathrm{T}\mathrm{O}\mathrm{T}}\left(t\right)/n}$$

where *n* and *p* represent the degrees of freedom of elemental variables and performance variable, respectively. V_TOT_ denotes the total variance, and each variance component was normalized per degree of freedom in the corresponding space. Due to the values in ∆V being constrained within computational boundaries, the synergy index was log transformed (Fisher's *z*-transformation, ∆V_Z_) to prevent the ceiling effect and to enable statistical comparisons (Kim et al. 2018; Robert et al. 2008, 2009).

To estimate the onset of the synergy-drop, the first derivative of ΔVz was computed from the time series. The time point with the most negative slope was identified as the center of the dominant drop. A two-segment piecewise linear regression was then applied to the preceding portion of the signal to estimate the breakpoint corresponding to the transition into the descending segment (Muggeo 2003). The onset of the synergy-drop was defined as the earliest time point preceding this breakpoint at which sustained steepening of the signal began.

#### Linear model of motor variability during cyclic force production

According to the linear model originally proposed by Gutman et al. (1993), the net performance in time-series such as net force *F*(*t*), acquired in multi-trials by multiple elements, is decomposed into two components of variance, V_UCM_ and V_ORT_. The relationship between these components and *F*(t), as well as the derivative of it, *dF*(*t*)/*dt*, was also examined. Specifically, when *F*(t) is altered, the modified linear model (Friedman et al. 2009; Latash et al. 2002) delineated a linear relationship between two variance components and the net outcome (net finger force) by the multiple elements, such that V_UCM_ and V_ORT_ exhibit the linear relationship to *F*(*t*) and *dF*(*t*)/*dt*, respectively. The linear regression analysis was performed to estimate the coefficients (or parameters), *a*_1_, *c*_1_, *a*_2_, *b*_2_, and *c*_2_ (Eqs. 4 and 5).

4 $${V}_{UCM}= {a}_{1}\cdot {F}_{TOT}+{c}_{1}$$

5 $${V}_{ORT}= {a}_{2}\cdot {F}_{TOT}+{b}_{2}\cdot \left|d{F}_{TOT}/dt\right|+{c}_{2}$$

For each participant, the linear regression was performed separately on the four cycles (C_PRE_–C_POST_) and for both C_UP_ and C_DW_ within each frequency condition. Consequently, linear model coefficients were estimated for each participant, resulting in thirty-two sets of coefficients. Furthermore, V_UCM_ and V_ORT_ were represented as single time-series derived from the accepted cycles, while total force (*F*_*TOT*_) and force rate (*dF*(*t*)/*dt*) used for the regression were obtained from trial-averaged time-series constructed from the same accepted cycles.

### Statistics

In general, a standard parametric statistical approach was utilized for repeated-measures ANOVAs, with means and standard error (SE) values reported. In contrast, non-parametric Wilcoxon signed-rank tests were implemented for the purpose of conducting post-hoc pairwise comparisons, with the Bonferroni *p*-value correction.

The performance outcome variables comprised F_ER_ and MPF. Finger force coordination outcome variables consisted of V_UCM_, V_ORT_, and ΔV_Z_, along with five coefficients (*a*_1_, *c*_1_, *a*_2_, *b*_2_, and *c*_2_) derived from the linear model. The statistical analyses were performed across several factors, including *Frequency* (four levels: LH, HL, LL, and HH), *Cycle* (four levels: C_PRE_, C_PRE-TRAN_, C_POST-TRAN_, and C_POST_), *C-Phase* (two levels: C_UP_ and C_DW_), *Extrema* (two levels: peak and trough), and *Variance* (two levels: UCM and ORT). Depending on the outcome variable, relevant factors were selected for the particular statistical tests. Specifically, F_ER_ was analyzed using a three-way repeated-measures ANOVA (*Frequency* × *Cycle* × *Extrema*), MPF using a two-way repeated-measures ANOVA (*Frequency* × *Cycle*), and the linear-model coefficients using a two-way repeated-measures ANOVA (*Frequency* × *Cycle*) conducted separately for the C_UP_ and C_DW_ phases. Time-series synergy variables (V_UCM_, V_ORT_, and ΔV_Z_) were examined using one-dimensional statistical parametric mapping (SPM).

Mauchly's sphericity test was used to confirm or reject the assumptions of sphericity. In instances where the sphericity assumption was rejected, the Greenhouse–Geisser correction was implemented. Furthermore, the effect sizes, partial eta squared (*ηp*^2^), and Cohen's *d*, were reported for all the presented results. Specifically, *ηp*^2^ was employed for ANOVA tests, while Cohen's *d* was used for *t*-tests (Cohen 2013). The statistical significance was set at *p* < 0.05.

To assess the impact of specific factors on the time-series variables derived from the UCM computation, one-dimensional statistical parametric maps (SPM) with repeated-measures ANOVAs (Pataky 2016) were employed. The SPM analyses were conducted using open-source software (Pataky 2012). A critical threshold for the SPM analyses was computed based on the random field theory (Worsley et al. 2004), which was set at α = 0.05. The SPM curve’s intersection with the critical threshold was identified as a significant effect of a particular factor during a designated time period of the curves. In instances where substantial effects pertaining to specific factors were observed, subsequent SPM{*t*} paired *t*-tests were conducted.

## Results

The terms, which were previously employed to denote a particular variable or condition within the description, bear a certain degree of similarity and may potentially lead to confusion. Therefore, emphasis is placed on reiterating the meaning of each term utilized in the Results Section 1) **Trial**: the execution of multiple cyclic force productions in 20 s. 2) **Cycle**: four distinct, isolated cycles, denoting C_PRE_–C_POST_, within a trial. 3) **Phase**: the phases of flexion force increase (C_UP_) and decrease (C_DW_) are distinguished within each of the four cycles. 4) **Frequency**: the target frequency conditions (LH, HL, LL, and HH). 5) **Extrema**: the peak and trough points within each cycle.

### Performance indices

#### Performance error in force magnitude (FER) during cyclic force production

**Fig. 3:**
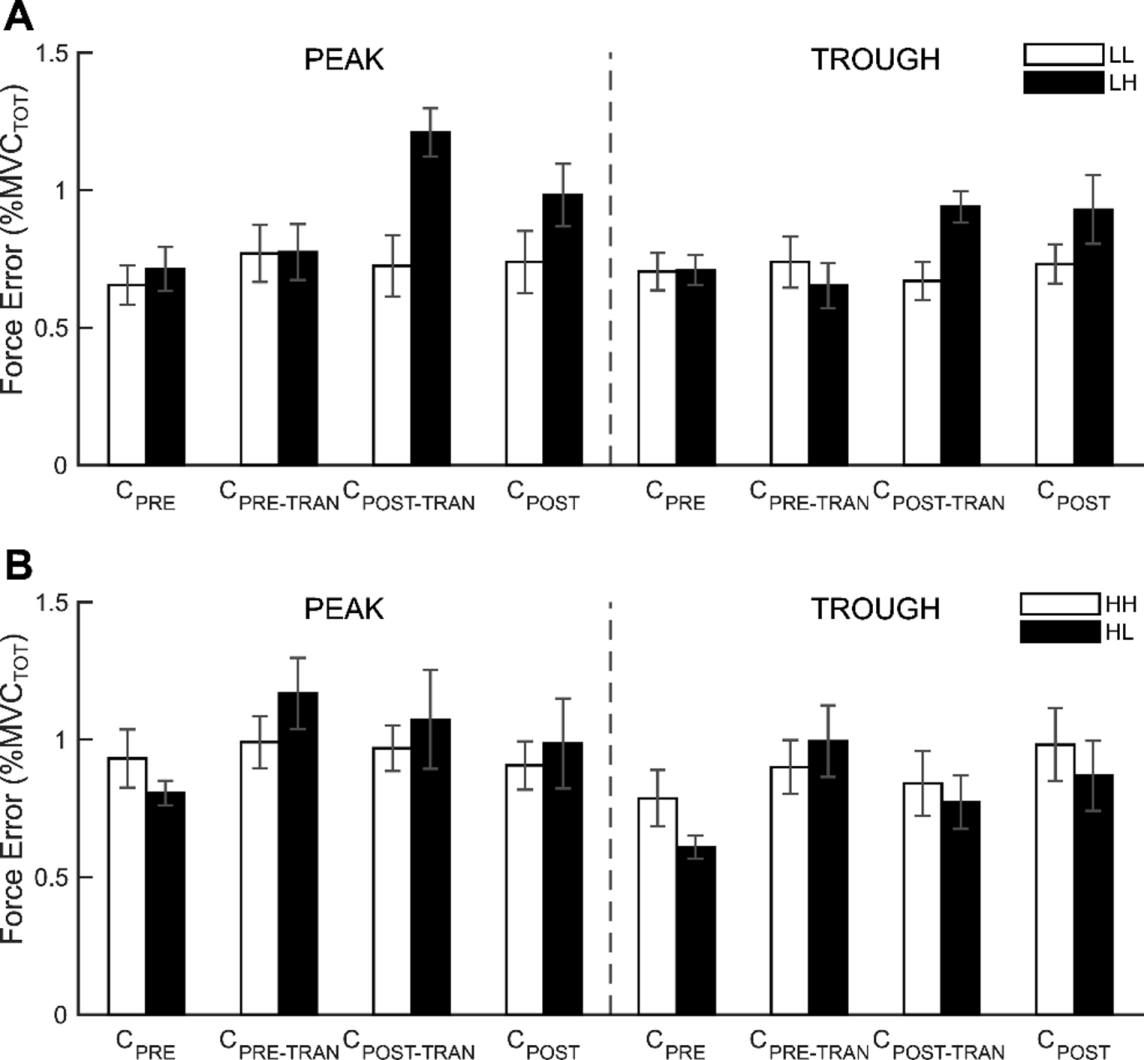
Force error during cyclic force production. **A** Force error at the peak and trough for the LL (white bars) and LH (black bars) conditions. **B** Force error at the peak and trough for the HH (white bars) and HL (black bars) conditions. Bars represent mean force error across participants for each of the four cycles (C_PRE_*–*C_POST_), and error bars indicate standard errors

The absolute magnitude of force error (F_ER_) did not exceed 1.5% of the MVC_TOT_ on average. The most salient observation was that the F_ER_ of both the peak and trough values appeared to be increased in the LH and HL conditions relative to those in the LL and HH conditions, particularly during C_PRE-TRAN_ or C_POST-TRAN_ across multiple cycles. No substantial alterations were observed in the F_ER_ of both the peak and trough in the LL and HH conditions, where no changes in frequency were required during the transition. The findings were corroborated by a three-way repeated-measures ANOVA with factors of *Frequency* (four levels: LH, HL, LL, and HH), *Cycle* (four levels: C_PRE_, C_PRE-TRAN_, C_POST-TRAN_, and C_POST_), and *Extrema* (two levels: peak and trough). The statistical outcome revealed a significant main effect of *Cycle* (*F*_[3,27]_ = 6.959, *p* = 0.001, *ηp*^2^ = 0.436) with a significant two-way factor interaction *Frequency* × *Cycle* (*F*_[2.49,22.42]_ = 3.732, *p* = 0.032, *ηp*^2^ = 0.293). The significant *Frequency* × *Cycle* interaction indicated that differences in F_ER_ across cycles were primarily observed in the LH and HL conditions, whereas F_ER_ remained relatively stable across cycles in the LL and HH conditions. Bonferroni-corrected post-hoc tests confirmed that the F_ER_ increased at C_POST-TRAN_ relative to C_PRE_ and C_PRE-TRAN_ in the LH condition (*p* = 0.019, *d* = 1.27; *p* = 0.027, *d* = 1.19), whereas in the HL condition, the F_ER_ was higher at C_PRE-TRAN_ than at C_PRE_ (*p* = 0.008, *d* = 1.46) (Fig. 3) .

#### Mean power frequency (MPF) during cyclic force production

**Fig. 4:**
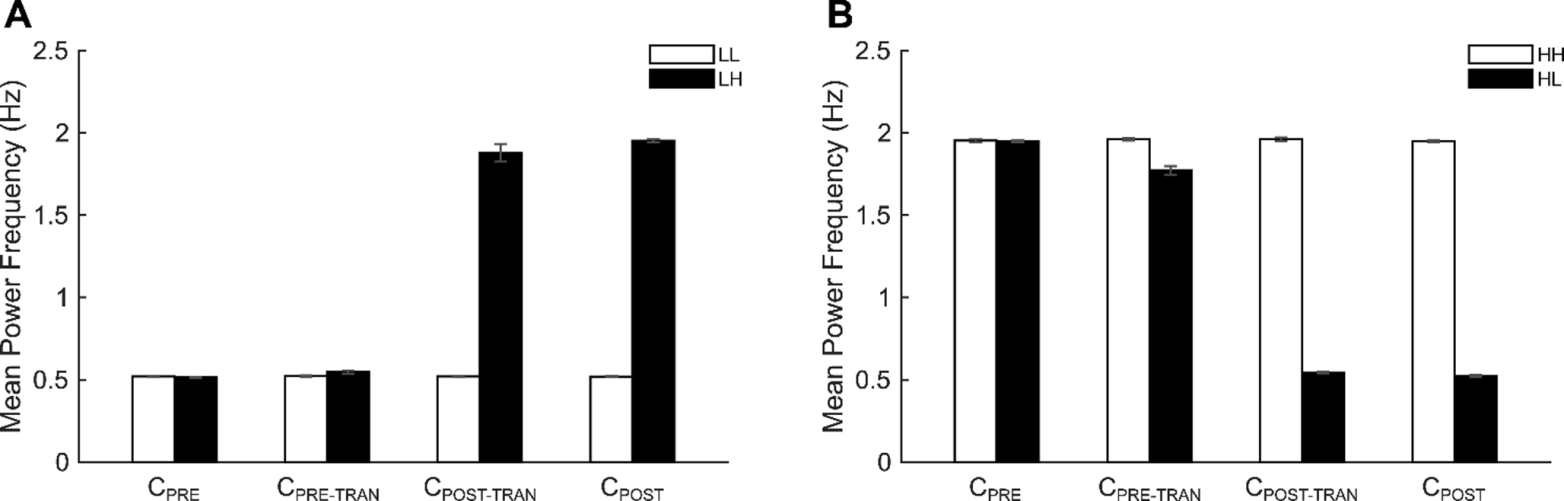
Mean power frequency (MPF). **A** MPF values for the LL (white bars) and LH (black bars) conditions. **B** MPF values for the HH (white bars) and HL (black bars) conditions. Bars represent mean values across participants for each of the four cycles (C_PRE_*–*C_POST_), and error bars indicate standard errors

The calculation of the MPF for each cycle was performed through the integration of the entire cycle, with the incorporation of the combination of C_UP_ and C_DW_. In general, the participants demonstrated a satisfactory degree of proficiency in executing the task, exhibiting a reasonable degree of accuracy in accordance with the target frequencies of cyclic force production. Specifically, the MPF in the LL and HH conditions exhibited a close match to the target frequencies, while the MPFs in the LH and HL conditions demonstrated slight deviations from the target frequency in specific cycles. The actual frequency (i.e., MPF) was lower than the target frequency, particularly in the LH and HL conditions at the cycles of the C_POST-TRAN_ and of C_PRE-TRAN,_ respectively, where the target frequency was 2 Hz (Fig. 4).

These observations were supported by two-way repeated-measures ANOVA with factors of *Frequency* (four levels: LH, HL, LL, and HH) and *Cycle* (four levels: C_PRE_, C_PRE-TRAN_, C_POST-TRAN_, and C_POST_). The analysis revealed significant main effects of *Frequency* (*F*_[1.06,13.55]_ = 5119.822, *p* < 0.001, *ηp*^2^ = 0.998) and *Cycle* (*F*_[1.52,13.68]_ = 5.536, *p* = 0.023, *ηp*^2^ = 0.381), as well as a significant *Frequency* × *Cycle* interaction (*F*_[1.63,14.70]_ = 1571.113, *p* < 0.001, *ηp*^2^ = 0.994). Bonferroni-corrected post-hoc tests showed that cycles performed at different target frequencies differed significantly. Among comparisons between cycles performed at the same target frequency, a significant difference was observed only in the HL condition, where the MPF at C_PRE-TRAN_ was lower than at C_PRE_ (*p* < 0.001).

### Multi-finger coordination indices

#### Changes in two components of variances and synergy indices during cyclic force production

**Fig. 5:**
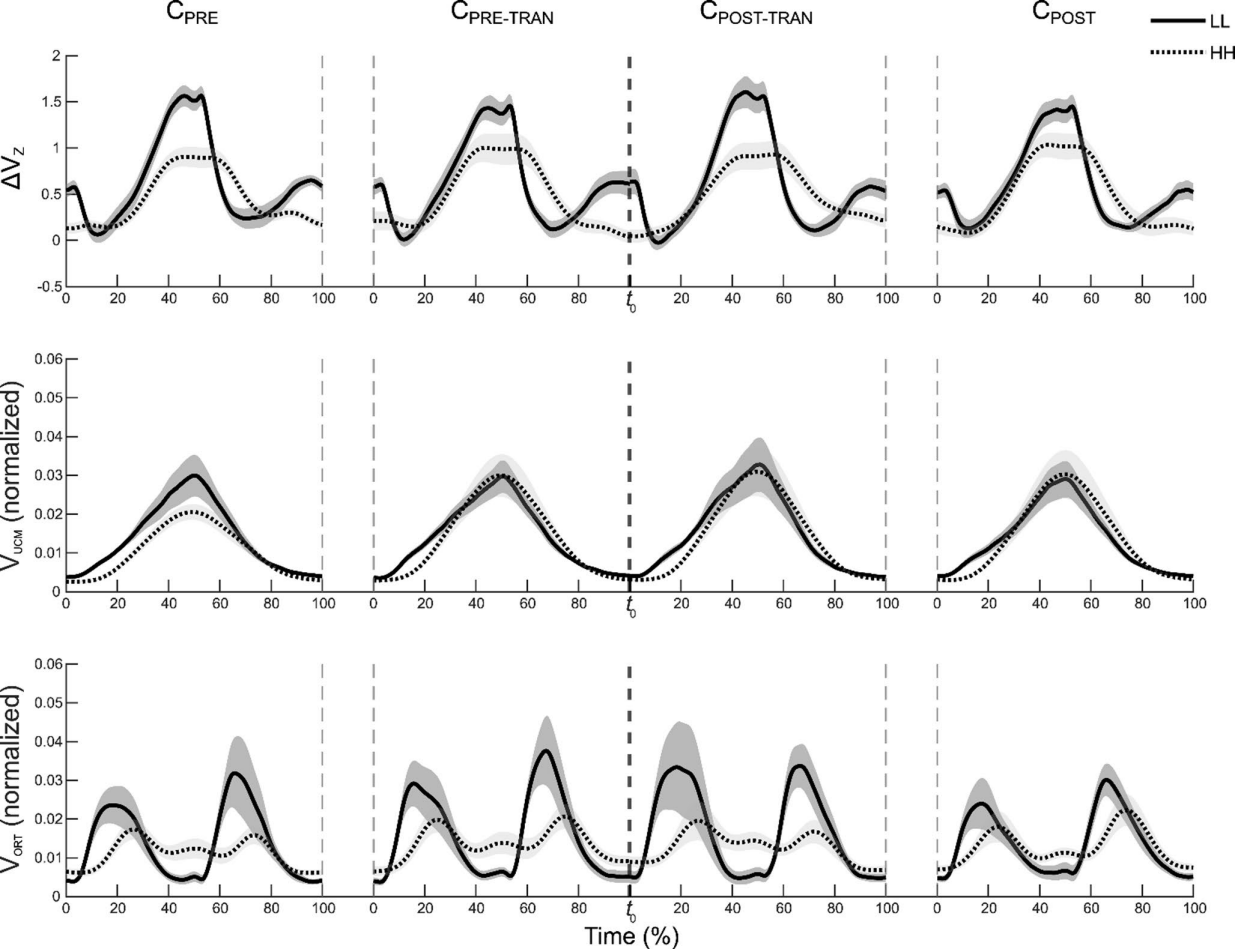
Time-series of ΔV_Z_ (synergy index), V_UCM_ (UCM variance), and V_ORT_ (ORT variance) in the LL and HH conditions without a frequency transition. Panels show ΔV_Z_ (top row), V_UCM_ (middle row), and V_ORT_ (bottom row) for the four cycles (C_PRE_–C_POST_) in the LL (solid line) and HH (dotted line) conditions. Lines represent mean values across participants, and shaded areas indicate standard errors. The vertical dashed line denotes the frequency transition time point (*t*_0_), although no frequency transition occurred in the LL and HH conditions. Time is normalized to the cycle duration and expressed as a percentage of the cycle (0–100%)

**Fig. 6:**
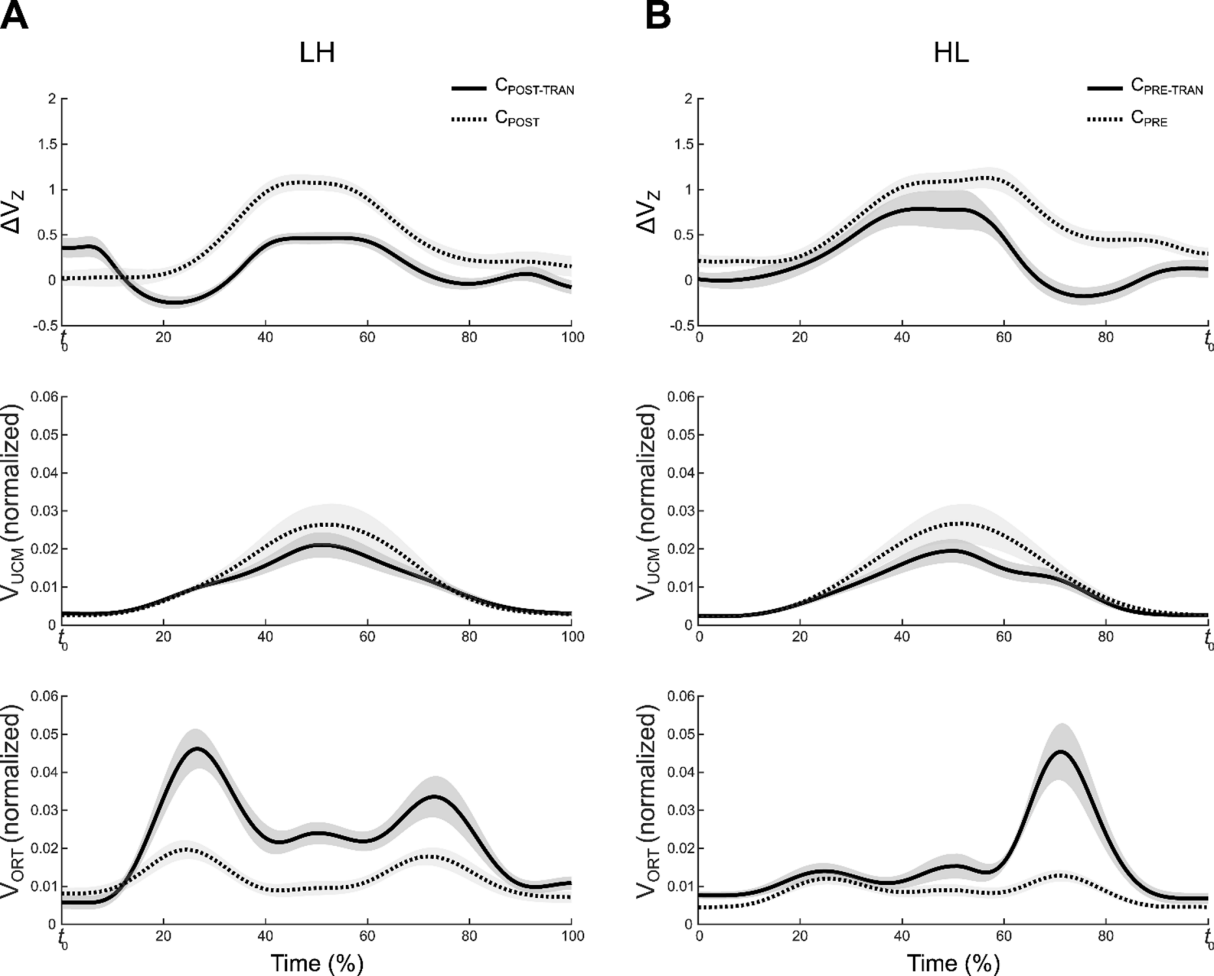
Time-series of ΔV_Z_ (synergy index), V_UCM_ (UCM variance), and V_ORT_ (ORT variance) in the LH and HL conditions. Time is normalized to the cycle duration and expressed as a percentage of the cycle (0–100%). **A** Comparison of the C_POST-TRAN_ and C_POST_ cycles in the LH condition. Solid lines denote the C_POST-TRAN_ cycle, and dotted lines denote the C_POST_ cycle. **B** Comparison of the C_PRE-TRAN_ and C_PRE_ cycles in the HL condition. Solid lines denote the C_PRE-TRAN_ cycle, and dotted lines denote the C_PRE_ cycle. It should be noted that not all cycle pairs were presented in Figure; rather, only those pairs for which distinct differences were observed were included, given that the majority of cycle pairs exhibited comparable patterns. In both panels, lines represent mean values across participants, and shaded areas indicate standard errors

The averaged time-series profiles of V_UCM_, V_ORT_, and ΔV_Z_ (synergy index) across participants are presented in Figs. 5 and 6. For the LL and HH (Fig. 5) conditions, V_UCM_, V_ORT_, and ΔV_Z_ delineated analogous patterns across all four cycles. A number of discrepancies were identified between the two frequency conditions: two local minima (troughs) were observed in ΔV_Z,_ and two sharp peak points of V_ORT_ in the LL condition. The HH condition demonstrated no clear local minima of ΔV_Z,_ a shallow peak, and a smaller range of fluctuation of V_ORT_ values. To provide a clearer description of the changes in variances and synergy index in the HL (Fig. 6B) and LH (Fig. 6A) conditions, comparisons focused on cycles performed at the same target frequency within each condition: C_PRE_ vs. C_PRE-TRAN_ in the HL condition and C_POST-TRAN_ vs. C_POST_ in the LH condition. As illustrated in Fig. 6, only specific cycle pairs were presented (specifically, C_POST_ and C_POST-TRAN_ for the LH condition, and C_PRE_ and C_PRE-TRAN_ for the HL condition) where distinct differences were observed. The rationale behind this selection is that other cycle pairs exhibited a comparable pattern. Consequently, not all cycle pairs were emphasized in the figure.

In the HL condition, the first pair of cycles (C_PRE_ and C_PRE-TRAN_) was performed at 2 Hz, whereas the latter pair (C_POST-TRAN_ and C_POST_) was performed at 0.5 Hz. The latter pair showed similar patterns across two cycles. In contrast, C_PRE-TRAN_ differed markedly from C_PRE_. Specifically, V_ORT_ in C_PRE-TRAN_ showed a clear peak during the C_DW_ phase, accompanied by a pronounced drop in ΔV_Z_ within the same phase. V_UCM_ remained relatively stable across these two cycles.

In the LH condition, the first pair of cycles (C_PRE_ and C_PRE-TRAN_), performed at 0.5 Hz, showed comparable patterns in V_UCM_, V_ORT_, and ΔV_Z_. In contrast, the latter pair (C_POST-TRAN_ and C_POST_), performed at 2 Hz, displayed clear differences. V_ORT_ in C_POST-TRAN_ exhibited a distinct peak during the C_UP_ phase, accompanied by a pronounced drop in ΔV_Z_ within the same phase, while V_UCM_ remained relatively consistent across the two cycles.

These observations were supported by SPM-based repeated-measures ANOVAs, which were conducted separately for each variance component (V_ORT_, V_UCM,_ and ΔV_Z_) and for each frequency condition (LH, HL, LL, and HH) to examine the effect of *Cycle* (four levels: C_PRE_, C_PRE-TRAN_, C_POST-TRAN_, and C_POST_). Significant clusters in V_UCM_, V_ORT_, and ΔV_Z_ from the SPM{F} analyses were primarily observed in the LH and HL conditions. Specifically, only clusters confirmed by Bonferroni-corrected post-hoc SPM{t} tests for cycle pairs sharing the same target frequency were interpreted. In the LH condition, pairwise comparisons between C_POST-TRAN_ and C_POST_ revealed significant clusters in V_ORT_ and ΔV_Z_ (approximately 20–40% and 20–60% of the cycle, respectively; both *p* < 0.001). In contrast, in the HL condition, comparisons between C_PRE_ and C_PRE-TRAN_ showed significant clusters only in ΔV_Z_ (approximately 55–85% of the cycle; *p* < 0.001).

#### Changes in coefficients of linear model by feedforward adjustment

Five coefficients (*a*_1_, *c*_1_, *a*_2_, *b*_2_, and *c*_2_) were obtained from the linear model. The parameters *a*_1_ and *c*_1_ represented the dependence of V_UCM_ on total force (F_TOT_) and its baseline offset, respectively. The parameters *a*_2_ and *b*_2_ represented the dependence of V_ORT_ on F_TOT_ and the absolute value of its time derivative (|*dF*/*dt*|), respectively, while *c*_2_ represented the baseline component of V_ORT_ variance. Two-way repeated-measures ANOVAs with factors *Frequency* (LH, HL, LL, HH) and *Cycle* (C_PRE_, C_PRE-TRAN_, C_POST-TRAN_, C_POST_) were conducted separately for the C_UP_ and C_DW_ phases for each coefficient. Significant main effects and interactions were observed only for the coefficients associated with V_ORT_ (*a*_2_, *b*_2_, and *c*_2_), whereas no significant effects were found for the coefficients related to V_UCM_ (*a*_1_ and *c*_1_).

The coefficients related to V_UCM_ (*a*_1_ and *c*_1_) did not show substantial variation across frequency or cycle conditions in either C_UP_ or C_DW_ phases, indicating that the relationship between V_UCM_ and F_TOT_ remained relatively stable throughout the task.

In contrast, the coefficients related to V_ORT_ showed marked modulation around the transition cycles. The coefficient *a*_2_ increased at C_POST-TRAN_ in the LH condition, particularly in the C_DW_ phase, while the C_UP_ phase showed a similar but less pronounced pattern. Across all frequency conditions, *b*_2_ showed higher values during the 0.5-Hz target cycles and lower values during the 2-Hz target cycles. Within this pattern, *b*_2_ increased markedly at C_POST-TRAN_ in C_UP_ under the LH condition and at C_PRE-TRAN_ in C_DW_ under the HL condition. The intercept *c*_2_ tended to show negative values during the 0.5-Hz target cycles and positive values during the 2-Hz target cycles across all frequency conditions Tables 1 and 2.

**Table 1:** Linear regression coefficients across cycle and frequency conditions in a representative participant. All linear regressions were statistically significant (*p* < 0.001)

Frequency	LL				LH				HH				HL			
Cycle	C_PRE_	C_PRE-TRAN_	C_POST-TRAN_	C_POST_	C_PRE_	C_PRE-TRAN_	C_POST-TRAN_	C_POST_	C_PRE_	C_PRE-TRAN_	C_POST-TRAN_	C_POST_	C_PRE_	C_PRE-TRAN_	C_POST-TRAN_	C_POST_
*V*_*UCM*_*, Force increase*																
R^2^	0.95	0.99	0.92	0.93	0.93	0.95	0.99	0.96	0.97	0.95	0.93	1.00	0.96	0.99	0.81	0.58
*a*_1_(× 10^4^)	17.44	18.84	26.88	16.59	6.32	11.23	14.28	12.25	13.47	14.30	8.33	10.88	6.45	8.16	4.24	3.06
*c*_1_ (× 10^4^)	−11.88	−27.31	−68.73	−20.34	18.08	14.43	−23.49	−3.63	−5.34	12.91	12.24	14.89	17.59	7.20	12.70	40.22
*V*_*UCM*_*, Force decrease*																
R^2^	0.96	0.87	0.90	0.84	0.95	0.98	0.97	0.99	0.89	0.86	0.68	0.81	0.99	0.92	0.83	0.94
*a*_1_(× 10^4^)	16.93	16.91	22.64	14.68	5.41	17.71	14.78	13.24	7.40	8.25	4.20	8.35	5.20	6.69	5.05	3.16
*c*_1_ (× 10^4^)	−28.57	−18.63	−59.44	−14.79	42.48	−36.25	−39.75	−1.94	26.72	22.45	33.12	16.03	23.87	4.93	−0.35	27.51
*V*_*ORT*_*, Force increase*																
R^2^	0.55	0.47	0.93	0.81	0.85	0.64	0.77	0.78	0.85	0.66	0.67	0.82	0.81	0.76	0.46	0.13
*a*_2_(× 10^4^)	0.79	0.21	1.50	−1.84	−1.49	1.37	1.33	−2.18	4.95	5.08	1.74	0.95	2.81	5.50	−6.98	7.37
*b*_2_(× 10^4^)	6.03	3.83	18.12	6.54	11.10	4.57	27.69	6.17	4.99	0.67	3.19	18.09	7.23	3.45	10.27	8.84
*c*_2_(× 10^4^)	−0.26	14.17	−73.84	11.42	−9.69	11.81	−66.62	40.47	28.79	35.98	80.26	2.83	12.72	74.14	143.99	19.31
*V*_*ORT*_*, Force decrease*																
R^2^	0.68	0.62	0.91	0.31	0.58	0.69	0.43	0.83	0.76	0.59	0.10	0.68	0.79	0.67	0.69	0.51
*a*_2_(× 10^4^)	−1.37	−4.57	−0.24	−5.11	−4.65	−7.12	1.72	−1.27	4.91	−1.57	1.11	−0.69	−0.54	−2.45	−4.66	−1.80
*b*_2_(× 10^4^)	5.97	10.81	8.59	4.23	9.07	17.04	2.45	4.05	3.11	3.90	0.56	8.18	4.86	10.72	16.68	6.57
*c*_2_(× 10^4^)	2.52	10.42	−17.12	63.10	19.41	−3.18	75.56	50.47	43.86	105.04	79.89	60.05	50.45	75.12	−20.51	25.40

**Table 2:** Results of two-way repeated-measures ANOVAs on linear model coefficients. Significant effects are indicated as *p* < 0.05 (*), *p* < 0.01 (**), and *p* < 0.001 (***)

Dependent variables		Factor	df	F	*p*
*a*_1_	C_UP_	*Frequency*	1.513,13.621	2.094	0.167
		*Cycle*	3,27	0.065	0.978
		*Frequency* × *Cycle*	2.979,26.812	1.737	0.184
	C_DW_	*Frequency*	1.317,11.855	1.463	0.265
		*Cycle*	3,27	0.131	0.941
		*Frequency* × *Cycle*	2.779,25.012	2.101	0.129
*c*_1_	C_UP_	*Frequency*	1.626,14.634	1.337	0.283
		*Cycle*	3,27	0.642	0.594
		*Frequency* × *Cycle*	2.900,26,098	1.148	0.340
	C_DW_	*Frequency*	3,27	1.751	0.180
		*Cycle*	3,27	0.160	0.922
		*Frequency* × *Cycle*	3.783,34.041	1.712	0.173
*a*_2_	C_UP_	*Frequency*	3,27	0.232	0.873
		*Cycle*	3,27	0.835	0.486
		*Frequency* × *Cycle*	3.130,28.170	1.945	0.143
	C_DW_	*Frequency*	3,27	4.692	** < 0.01** ^**^
		*Cycle*	3,27	6.207	** < 0.01** ^**^
		*Frequency* × *Cycle*	3.315,29.832	3.169	** < 0.05** ^*^
*b*_2_	C_UP_	*Frequency*	3,27	8.490	** < 0.001** ^***^
		*Cycle*	3,27	7.218	** < 0.01** ^**^
		*Frequency* × *Cycle*	9,81	3.503	** < 0.01** ^**^
	C_DW_	*Frequency*	3,27	12.859	** < 0.001** ^***^
		*Cycle*	3,27	2.943	0.051
		*Frequency* × *Cycle*	9,81	4.193	** < 0.001** ^***^
*c*_2_	C_UP_	*Frequency*	3,27	13.526	** < 0.001** ^***^
		*Cycle*	3,27	2.505	0.080
		*Frequency* × *Cycle*	9,81	4.118	** < 0.001** ^***^
	C_DW_	*Frequency*	1.493,13.437	5.060	** < 0.05** ^*^
		*Cycle*	3,27	3.091	** < 0.05** ^*^
		*Frequency* × *Cycle*	2.893,26.034	2.351	0.097

## Discussion

The second and third hypotheses formulated in the Introduction were substantiated by the observation that the primary component of synergy-drop prior to the alternation of frequency of cyclic force was the error variance, V_ORT_, while V_UCM_ exhibited minimal change across the conditions in comparison to the pronounced alternation in the V_ORT_. Additionally, the anticipatory synergy adjustment was not clearly observed in the absence of changes in the frequencies of the cyclic force, LL and HH conditions.

Conversely, our prediction, the first hypothesis concerning the prevalence of the purposeful destabilization, was not fully supported by the experimental outcomes. The findings indicated that the decline in the synergy index prior to the alternation in frequency was more pronounced when the frequency of cyclic force transited from a high to a low state, as opposed to the low to high transition, which contradicted our initial hypothesis. In the Discussion, we explore the implications of these findings with respect to the intricate mechanism of feedforward adjustment and its beneficial consequence, encompassing alternation in the two components of variance and the frequency-dependency of the anticipation strategies.

### Synergy-drop as evidence of feedforward changes in human motor system: circumstance of utilization of feedforward adjustment of synergy index

A salient finding of the present study was that the decline in the synergy indices, given the abundance of DoFs present in the human body system, may be a pervasive phenomenon even in the adjustment strategy to imminent changes in dynamics. In particular, the activation of the feedforward mechanism is contingent upon the sequence of changes in the dynamics of the tasks, e.g., HL and LH conditions in the present experiment. In contrast to the HL and LH conditions, the absence of pattern changes in the synergy indices in the LL and HH conditions, which did not require alternation in the frequencies, may serve as counter-evidence to the prerequisite of the activation of the feedforward mechanism in human behaviors, such as the changes in covariation patterns of elements to the new motor tasks. The visual structures under investigation were found to be consistent in both the LH and HL conditions, indicating that the subjects possessed equivalent knowledge of the impending transition in both cases. The key difference between the LH and HL conditions was not attributable to the presence of visual information; rather, it was attributed to the task dynamics that encompassed the subsequent action following the crossing of the second vertical line. In the context of these two conditions, the preservation of existing synergy would be functionally appropriate, suggesting that there is minimal justification in the perspective of motor control for weakening the existing force-stabilization synergy. This interpretation is consistent with both the pulse-force and cyclic force production supported by prior findings (De et al. 2024; Latash et al. 2002), which demonstrated that the fundamental synergy modulation is evident when demands for alternation in speed or force arise in anticipation of subsequent tasks. In particular, De et al. (2024) revealed that transient ASA scaled with the speed of the forthcoming action during the discrete force production task. This finding was opposite to the results of the present findings regarding the speed of impending action. In the present cyclic paradigm, the future action speed and available preparatory time were dissociated. The first hypothesis of the present study was formulated on the basis of the estimated time duration difference between the high-to-low (HL) and the low-to-high (LH) conditions. This phenomenon can be attributed to the time sensitivity of the observation, which encompasses the delayed time of adjustment observed in elderly group and neurological disorder patients (Olafsdottir et al. 2007a; Park et al. 2012). The hypothesis is predicated on the assumption that the duration of time was inadequate for the activation of the feedforward mechanism in the HL condition, and vice versa for the LH condition. Contrary to prediction, the synergy-drop phenomenon was distinctly observed earlier in the high-to-low (HL) condition, whereas it was delayed in the low-to-high (LH) condition, as evidenced by the analysis. Therefore, the present finding indicates that, in cyclic frequency-transition tasks, the observable expression of ASA is influenced not only by future action speed but also by the temporal resources available for reorganization within the ongoing cycle. In this sense, the present data dissociate two influences that are coupled in discrete force-change paradigms: future action speed appears to affect the required magnitude or cost of destabilization, whereas preparation-time pressure appears to affect when the synergy drop becomes detectable.

### The strategies of synergy-drop as feedforward mechanism: strategy of synergy adjustment that occurs when forthcoming frequency of cyclic force increases

The additional comparable discrepancy between the present and previous studies was observed in the primary component of variance for the synergy-drop, which exhibited an opposite nature between the discrete (Friedman et al. 2009; Kim et al. 2006; Latash et al. 2002; Olafsdottir et al. 2005; Park et al. 2012) and the present cyclic force production, with alterations in V_UCM_ for the quick pulse and V_ORT_ for cyclic force production. This discrepancy between the present cyclic task and previously studied quick-pulse paradigms may reflect a fundamental distinction between rhythmic and discrete motor actions. According to the extant literature, discrete and rhythmic movements are hypothesized to be governed by disparate neural and behavioral control processes (see Buchanan et al. 2004; Guiard 1997; Hogan and Sternad 2007; Schaal et al. 2004). In this framework, the observed variance structure in the present cyclic task may partly reflect control strategies associated with rhythmic movement generation rather than those typically observed in discrete actions. Consistent with this perspective, the results showed distinct patterns between the HL and LH conditions. It should be noted that the synergy-drop phenomenon was also observed in the LH condition; however, the destabilization occurred after the predetermined time for frequency modification. A notable finding was the substantial decrease in the synergy index concomitant with a considerable increase in V_ORT_ at the onset of the pre-transition cycle in the HL condition (C_PRE-TRAN_ in Fig. 6B) and post-transition cycle in the LH condition (C_POST-TRAN_ in Fig. 6A), which indicates a distinctive difference in the phenomenon of the synergy-drop between the two conditions. We estimated the average actual time of the initiation of the synergy-drop, not the normalized percent time shown in Fig. 6. The mean value for the HL condition was approximately − 255 ms, while the mean value for the LH condition was + 30 ms, on average across participants, with respect to the predetermined time (*t*_0_) for the alternation of the frequency in the cyclic force production. It is noteworthy that the time point of the synergy-drop in the HL condition corresponded to the conventional time window of the anticipatory synergy adjustment from different experimental paradigms (Jo et al. 2016; Park et al. 2012; Piscitelli et al. 2017; Zhou et al. 2013). In particular, the adjustment (synergy-drop) commenced instantaneously at the beginning of C_PRE-TRAN_, occurring − 250 ms into the given time duration for a single cycle at 2 Hz. The explicit instruction provided to the participants was to generate the same frequency cycles by hearing metronome beats until the feedback cursor passed the vertical line, denoting a change in the frequency of the cyclic force. It appears that all participants adhere to the given instruction consistently for all conditions, including the high-to-low (HL) condition.

The first hypothesis did not sufficiently account for the sensory context of the present task. Because real-time force feedback was continuously provided to the participant during the trial, visual information should be considered as part of the control architecture of the present cyclic force production. Participants received continuous visual feedback of the total force produced, with the second vertical denoting the constrained time of the frequency transition. The metronome provided the temporal structure of the required cyclic rhythm. i.e., the timing of the peak and trough force production. Consequently, the present task did not depend solely on an internal timing process. At the same time, visual anticipation of the transition line is unlikely to explain the HL–LH asymmetry by itself, because the visual display was identical across the two transition conditions. Furthermore, with the exception of the very first cycle, a continuous target waveform (force template) was not presented; rather, participants primarily observed their own produced force trace in real time, whereas the metronome assisted in regulating the timing of peak and trough force production. Therefore, visual information is interpreted here mainly as contributing to online monitoring and correction of the ongoing force waveform (Latash et al. 2005; Todorov and Jordan 2002), whereas the opposite timing in HL and LH is more plausibly related to direction-specific task demands, namely the dynamics of the forthcoming action and the time available for synergy reorganization within the ongoing cycle. Because visual feedback was not manipulated experimentally, the present data do not allow isolation of its modality-specific contribution, which would be the reason why the first hypothesis was not supported. This issue should be addressed in future work.

A remaining question concerns the LH condition, in which the detectable decrease in the synergy index occurred at approximately + 30 ms relative to *t*_*0*_. Relative to the prescribed transition time, this onset does not satisfy a strict classical definition of anticipatory synergy adjustment. However, the present behavioral data also do not justify attribution of this effect to a specific physiological feedback pathway. Participants knew the transition timing in advance and received continuous sensory information regarding ongoing force output, and the present experiment did not include EMG, perturbation probes, or other physiological measures. Accordingly, the LH pattern is interpreted here more conservatively as a delayed or boundary-crossing manifestation of predictive synergy modulation, possibly accompanied by ongoing online correction. This interpretation is also consistent with the variance structure of the present results: the reduction in ΔV was expressed mainly through increased V_ORT_, whereas V_UCM_ remained relatively unchanged. Thus, the larger V_ORT_ near the transition is interpreted as a transition-related coordination cost, rather than as an ASA-specific marker by itself. In contrast, the HL condition, with an onset around − 255 ms relative *t*_*0*_, more closely matched the conventional ASA time window. However, given the limitations of the current experimental configuration and measured data, it is imperative that future studies employ electromyography (EMG) or transcranial magnetic stimulation (TMS) metrics to substantiate the validity of the current claims. The methodologies of the EMG and TMS are complementary, such that the EMG is a technique that can be used to measure the timing and pattern of muscle activation (Danna-Dos-Santos et al. 2007; Weiler et al. 2015), while the TMS is another technique that can be used to detect the involvement of the corticospinal tract and the primary motor cortex (M1) (Chiou et al. 2018; Niu et al. 2008). In this regard, the metrics measured by the two methods would provide strong physiological evidence regarding whether the current mechanical outcome and synergy formation and modulation are the neurophysiological-nature phenomenon.

### Functional and beneficial effect of feedforward adjustment during cyclic force production

A thorough examination of the performance indices, including the performance error and the satisfaction of the instructed frequency during the transition of the frequency of cyclic force, revealed no significant advantage. This finding was corroborated by the comparable force error and mean power frequency at four distinct cycles between the LH and HL conditions. Furthermore, the magnitudes of the synergy-drop between the two conditions were found to be comparable. However, a critical distinction emerged between the two conditions regarding the temporal adjustment of the synergy. Specifically, the specific cycle out of four cycles in which the synergy-drop was detected differed. A substantial corpus of experimentations has been dedicated to the study of anticipatory synergy adjustment, which has led to the elucidation of the characteristics of this phenomenon. For instance, the time of the anticipatory synergy adjustment was delayed in the elderly group (Olafsdottir et al. 2008) and various types of neurological patients (Jo et al. 2016; Park et al. 2012). Also, the extent of the destabilization (i.e., the magnitude of the decreased value of the synergy index) may be associated with the efficiency of the energy consumption, even with similar values of the synergy indices during the steady-state force production (Latash 2024). The maintenance of the synergy may require energy; thus, less drop of the synergy indices, i.e., keeping the synergy, during the dynamical circumstance, is not a beneficial strategy in a sense of physiology and motor control. Therefore, the synergy formation and its modulation are potentially two distinct neural processes (Kong et al. 2019). However, the advantageous aspect of the activation of the feedforward mechanism regarding the level of performance has been rarely reported. A recent study on simulated archery shooting has indicated that the accuracy and precision of performance were enhanced by releasing the force at a self-selected time that utilized the feedforward mechanism (Kim et al. 2024; Song et al. 2023). In this regard, the present study is concerned with ascertaining the advantageous aspects of the performance level that can be derived from the anticipatory synergy adjustment in light of the cyclic force production task, which is more ecologically relevant to everyday activities as compared to the quick pulse or release followed by the steady-state force production. In other words, the present task was simplified and isometric. It captures an important feature of everyday hand use: finger forces often have to be modulated continuously and predictively across repeated cycles, for example, when holding a cup or phone while walking or stepping down a stair, or when transporting a hand-held object during cyclic arm movements.

In this context, the control of cyclic force production necessarily involves precise regulation of both force magnitude and its rate of change across cycles. The duration of the cycle is determined by the frequency, but the frequency does not act as the sole determinant of the speed. The peak value of speed is determined by both the amplitude and frequency. In the present experiment, however, the amplitude of all the cyclic forces was identical across all the experimental conditions. Consequently, the changes in frequency also changed the required force-rate demand. Under these constraints, the peak rate of force change in the 2 Hz condition was four times larger than that in the 0.5 Hz condition. The time-varying function can be expressed as follows: |*dF*/*dt*|= 4·**f**·**A**, where **f** and **A** represent the frequency and amplitude, respectively. The present data do not permit direct separation of feedforward and feedback contributions. A more conservative interpretation is that the HL condition required earlier predictive reorganization because the available preparation interval within the ongoing cycle was brief, whereas in the LH condition, online correction remained more behaviorally available around the transition. From this perspective, the major difference between HL and LH is not the magnitude of the synergy drop, which was comparable between conditions, but the timing of its observable expression. Thus, the present findings suggest that cyclic force transitions are accommodated primarily through temporally specific reorganization of coordination, with increased task-relevant variance reflecting the cost of adjusting the ongoing waveform under changing force-rate demands. In essence, the predominance of feedforward control does not inherently signify a decline in control efficacy. Rather, it frequently indicates that the central nervous system (CNS) is prioritizing the regulation of the entire waveform by employing a time-parameterized factor, as opposed to focusing on minimizing errors at the point-by-point level. Consequently, it is plausible that the minor variability in the time-related parameter (i.e., inadequate time) projects into the orthogonal space to the null space, V_ORT_, when the rate of change of the force (*dF*/*dt*) is augmented.

## Conclusion

The present study examined anticipatory synergy adjustments during cyclic force production with frequency transitions. The results showed that the magnitude of the synergy-drop was comparable between conditions, whereas its timing differed between the HL and LH conditions. Specifically, earlier synergy-drop was observed in the HL condition, while it was delayed in the LH condition, indicating differences in the temporal organization of covariation patterns. This modulation was primarily driven by increases in V_ORT_. A main limitation of the present study is that the speed of the forthcoming action and the time available for anticipatory reorganization were coupled by the task design. Therefore, the present findings cannot determine whether the HL–LH asymmetry was driven by future task dynamics, time pressure within the ongoing cycle, or their interaction. In addition, because sensory information was continuously available and no physiological measures were collected, the contributions of predictive and reactive processes could not be isolated. Future studies should dissociate force-rate demands from preparation time, manipulate sensory feedback, and combine high-resolution force measures with EMG or TMS to clarify the mechanisms underlying synergy modulation. Overall, the findings suggest that synergy modulation during cyclic force transitions differed mainly in timing rather than magnitude, indicating temporally specific reorganization of multi-finger coordination under changing task dynamics.

## Data Availability

Will be made available upon a reasonable request.

## Appendix

### Uncontrolled manifold (UCM) computation

In the multi-finger force production task, changes in the total finger force (*dF*_TOT_) resulted from the changes in any of the individual finger forces: *df*, where *f* = [ *f *_i_ *f*_m_ *f*_r_ *f *_l_]^T^; T denotes a matrix transpose. Therefore, the Jacobian that represents the linear relationship between the forces of four individual fingers as input variables and total force as an output variable was defined as [1 1 1 1], as described by Eq. 6.

6 $$ dF_{{TOT}} (t) = \left[ {1111} \right] \cdot df(t) $$

The UCM space was defined as the null space of the Jacobian in the four-dimensional force space. In this space, flexible combinations of demeaned finger forces satisfy the constraint and therefore produce no change in the net force (Eq. 7).

7 $$ {0 = \left[ {1111} \right] \cdot e_{i} } $$

In the next step, the mean-free values were projected onto the null space (UCM space) and summed as described in Eq. 8.

8 $$ f(t) = \sum\limits_{i}^{{n - p}} {(e_{i}^{T} } \cdot df\left( t \right)) \cdot e_{i} $$

where *n* = 4, representing the dimensionality or degrees of freedom of the original force space. *p* = 1, signifying the number of constraints presented in the current motor task. The projection of data points onto the orthogonal complement of the null space was calculated as follows (Eq. 9).

9 $$ f_{ \bot } (t) = df(t) - f(t) $$

The subsequent computational step entailed the calculation of the variances in the UCM and its orthogonal complement (ORT) per DOF. Furthermore, within the UCM, the variance value was normalized by the 15% MVC_TOT_, corresponding to the target force level used in the task (Eq. 10).

10 $$ V_{{UCM}} (t) = \frac{{\sum {\left| {f(t)} \right|^{2} } }}{{\left( {n - p} \right) \cdot N_{{trials}} \cdot 0.15 \cdot MVC_{{TOT}} }} $$

The variance per DOF within the ORT space was calculated as shown in Eq. 11.

11 $$ V_{{ORT}} (t) = \frac{{\sum {\left| {f_{ \bot } (t)} \right|^{2} } }}{{p \cdot N_{{trials}} \cdot 0.15 \cdot MVC_{{TOT}} }} $$

The final step entailed the calculation of the synergy index (ΔV) in time-series format. The synergy index (ΔV) was defined as the normalized difference between V_UCM_ and V_ORT_ relative to the total variance (V_TOT_).

12 $$\begin{array}{c}\Delta V(t)=\frac{{V}_{\mathrm{U}\mathrm{C}\mathrm{M}}(t)/(n-p)-{V}_{\mathrm{O}\mathrm{R}\mathrm{T}}(t)/p}{{V}_{\mathrm{T}\mathrm{O}\mathrm{T}}(t)/n}\end{array}$$

The values in ∆V were constrained to the computational boundaries. Therefore, the log transform (Fisher's *z*-transformation, ∆V_Z_) was employed to circumvent the ceiling effect prior to statistical comparisons (Robert et al. 2008, 2009; Kim et al. 2018).

## References

[CR1] Aruin AS, Latash ML (1995) Directional specificity of postural muscles in feed-forward postural reactions during fast voluntary arm movements. Exp Brain Res 103:323–332. 10.1007/BF00231718 7789439 10.1007/BF00231718

[CR2] Bouisset S, Zattara M (1981) A sequence of postural movements precedes voluntary movement. Neurosci Lett 22(3):263–270. 10.1016/0304-3940(81)90117-8

[CR3] Bouisset S, Zattara M (1987) Biomechanical study of the programming of anticipatory postural adjustments associated with voluntary movement. J Biomech 20(8):735–742. 10.1016/0021-9290(87)90052-2 3654672 10.1016/0021-9290(87)90052-2

[CR4] Buchanan JJ, Park JH, Shea CH (2004) Systematic scaling of target width: dynamics, planning, and feedback. Neurosci Lett 367(3):317–322. 10.1016/j.neulet.2004.06.028 15337257 10.1016/j.neulet.2004.06.028

[CR5] Chiou SY, Hurry M, Reed T, Quek JX, Strutton PH (2018) Cortical contributions to anticipatory postural adjustments in the trunk. J Physiol 596(7):1295–1306. 10.1113/JP275312 29368403 10.1113/JP275312 PMC5878228

[CR6] Cohen J (2013) Statistical power analysis for the behavioral sciences, 2nd ed. Routledge, New York. 10.4324/9780203771587

[CR7] Danna-Dos-Santos A, Degani AM, Latash ML (2007) Anticipatory control of head posture. Clin Neurophysiol 118(8):1802–1814. 10.1016/j.clinph.2007.05.060 17581777 10.1016/j.clinph.2007.05.060 PMC2041881

[CR8] De SD, Ambike S, Latash ML (2024) Two aspects of feed-forward control of action stability: effects of action speed and unexpected events. Exp Brain Res 242:2177–2191. 10.1007/s00221-024-06892-x 38992203 10.1007/s00221-024-06892-x

[CR9] Flanagan JR, Wing AM (1997) The role of internal models in motion planning and control: evidence from grip force adjustments during movements of hand-held loads. J Neurosci 17(4):1519–1528. 10.1523/JNEUROSCI.17-04-01519.1997 9006993 10.1523/JNEUROSCI.17-04-01519.1997 PMC6793733

[CR10] Flanagan JR, Ostry DJ, Feldman AG (1993) Control of trajectory modifications in target-directed reaching. J Mot Behav 25(3):140–152. 10.1080/00222895.1993.9942045 12581985 10.1080/00222895.1993.9942045

[CR11] Friedman J, Skm V, Zatsiorsky VM, Latash ML (2009) The sources of two components of variance: an example of multifinger cyclic force production tasks at different frequencies. Exp Brain Res 196:263–277. 10.1007/s00221-009-1846-x 19468721 10.1007/s00221-009-1846-x PMC2745158

[CR12] Guiard Y (1997) Fitts’ law in the discrete vs. cyclical paradigm. Hum Mov Sci 16(1):97–131. 10.1016/S0167-9457(96)00045-0

[CR13] Gutman SR, Gottlieb GL (1992) Basic functions of variability of simple pre-planned movements. Biol Cybern 68:63–73. 10.1007/BF00203138 1486132 10.1007/BF00203138

[CR14] Gutman SR, Latash ML, Almeida GL, Gottlieb GL (1993) Kinematic description of variability of fast movements: analytical and experimental approaches. Biol Cybern 69:485–492. 10.1007/BF01185420 8274547

[CR15] Hogan N, Sternad D (2007) On rhythmic and discrete movements: reflections, definitions and implications for motor control. Exp Brain Res 181(1):13–30. 10.1007/s00221-007-0899-y 17530234 10.1007/s00221-007-0899-y

[CR16] Horak FB (2006) Postural orientation and equilibrium: what do we need to know about neural control of balance to prevent falls? Age Ageing 35(suppl_2):ii7–ii11. 10.1093/ageing/afl077 16926210 10.1093/ageing/afl077

[CR17] Ivanenko YP, Cappellini G, Dominici N, Poppele RE, Lacquaniti F (2005) Coordination of locomotion with voluntary movements in humans. J Neurosci 25:7238–7253. 10.1523/JNEUROSCI.1327-05.2005 16079406 10.1523/JNEUROSCI.1327-05.2005 PMC6725226

[CR18] Jo HJ, Maenza C, Good DC, Huang X, Park J, Sainburg RL, Latash ML (2016) Effects of unilateral stroke on multi-finger synergies and their feed-forward adjustments. Neuroscience 319:194–205. 10.1016/j.neuroscience.2016.01.054 26828408 10.1016/j.neuroscience.2016.01.054 PMC4770574

[CR19] Kim SW, Shim JK, Zatsiorsky VM, Latash ML (2006) Anticipatory adjustments of multi-finger synergies in preparation for self-triggered perturbations. Exp Brain Res 174:604–612. 10.1007/s00221-006-0505-8 16724179 10.1007/s00221-006-0505-8

[CR20] Kim K, Xu D, Park J (2018) Effect of kinetic degrees of freedom on multi-finger synergies and task performance during force production and release tasks. Sci Rep 8:12758. 10.1038/s41598-018-31136-8 30143688 10.1038/s41598-018-31136-8 PMC6109105

[CR21] Kim K, Song J, Park D, Park J (2024) Hierarchical organization and adjustment of force coordination in response to self-triggered and external-triggered cues in simulated archery performance. J Appl Biomech 40:323–332. 10.1123/jab.2022-0317 38942418 10.1123/jab.2022-0317

[CR22] Klous M, Mikulic P, Latash ML (2011) Two aspects of feedforward postural control: anticipatory postural adjustments and anticipatory synergy adjustments. J Neurophysiol 105(5):2275–2288. 10.1152/jn.00665.201 21389305 10.1152/jn.00665.2010 PMC3094180

[CR23] Kong J, Kim K, Joung HJ, Chung CY, Park J (2019) Effects of spastic cerebral palsy on multi-finger coordination during isometric force production tasks. Exp Brain Res 237:3281–3295. 10.1007/s00221-019-05671-3 31664488 10.1007/s00221-019-05671-3

[CR24] Latash ML (2024) Brief history of the Uncontrolled Manifold Hypothesis and its role in motor control. Braz J Mot Behav 18:e433. 10.20338/bjmb.v18i1.433

[CR25] Latash ML, Aruin AS, Neyman I, Nicholas J (1995) Anticipatory postural adjustments during self inflicted and predictable perturbations in Parkinson’s disease. J Neurol Neurosurg Psychiatry 58(3):326–334. 10.1136/jnnp.58.3.326 7897415 10.1136/jnnp.58.3.326 PMC1073370

[CR26] Latash ML, Scholz JF, Danion F, Schöner G (2001) Structure of motor variability in marginally redundant multifinger force production tasks. Exp Brain Res 141:153–165. 10.1007/s002210100861 11713627 10.1007/s002210100861

[CR27] Latash ML, Scholz JF, Danion F, Schöner G (2002) Finger coordination during discrete and oscillatory force production tasks. Exp Brain Res 146:419–432. 10.1007/s00221-002-1196-4 12355270 10.1007/s00221-002-1196-4

[CR28] Latash ML, Shim JK, Smilga AV, Zatsiorsky VM (2005) A central back-coupling hypothesis on the organization of motor synergies: a physical metaphor and a neural model. Biol Cybern 92:186–191. 10.1007/s00422-005-0548-0 15739110 10.1007/s00422-005-0548-0 PMC2827178

[CR29] Muggeo VM (2003) Estimating regression models with unknown break-points. Stat Med 22(19):3055–3071. 10.1002/sim.1545 12973787 10.1002/sim.1545

[CR30] Niu X, Zatsiorsky VM, Latash ML (2008) Stability of the multi-finger prehension synergy studied with transcranial magnetic stimulation. Exp Brain Res 190:225–238. 10.1007/s00221-008-1466-x 18592229 10.1007/s00221-008-1466-x PMC2561192

[CR31] Olafsdottir H, Yoshida N, Zatsiorsky VM, Latash ML (2005) Anticipatory covariation of finger forces during self-paced and reaction time force production. Neurosci Lett 381:92–96. 10.1016/j.neulet.2005.02.003 15882796 10.1016/j.neulet.2005.02.003 PMC2827154

[CR32] Olafsdottir H, Yoshida N, Zatsiorsky VM, Latash ML (2007a) Elderly show decreased adjustments of motor synergies in preparation to action. Clin Biomech 22:44–51. 10.1016/j.clinbiomech.2006.08.005 10.1016/j.clinbiomech.2006.08.005 PMC1829316 17046125

[CR33] Olafsdottir H, Zhang W, Zatsiorsky VM, Latash ML (2007b) Age-related changes in multifinger synergies in accurate moment of force production tasks. J Appl Physiol 102(4):1490–1501. 10.1152/japplphysiol.00966.2006 17204576 10.1152/japplphysiol.00966.2006 PMC2821089

[CR34] Olafsdottir H, Kim SW, Zatsiorsky VM, Latash ML (2008) Anticipatory synergy adjustments in preparation to self-triggered perturbations in elderly individuals. J Appl Biomech 24:175–179. 10.1123/jab.24.2.175 18579910 10.1123/jab.24.2.175 PMC2546357

[CR35] Oldfield RC (1971) The assessment and analysis of handedness: the Edinburgh inventory. Neuropsychologia 9:97–113. 10.1016/0028-3932(71)90067-4 5146491 10.1016/0028-3932(71)90067-4

[CR36] Park J, Wu Y-H, Lewis MM, Huang X, Latash ML (2012) Changes in multifinger interaction and coordination in Parkinson’s disease. J Neurophysiol 108:915–924. 10.1152/jn.00043.2012 22552184 10.1152/jn.00043.2012 PMC3424084

[CR37] Pataky TC (2012) One-dimensional statistical parametric mapping in Python. Comput Methods Biomech Biomed Eng 15:295–301. 10.1080/10255842.2010.527837 10.1080/10255842.2010.527837 21756121

[CR38] Pataky TC (2016) rft1d: smooth one-dimensional random field upcrossing probabilities in Python. J Stat Softw 71:1–22. 10.18637/jss.v071.i07

[CR39] Piscitelli D, Falaki A, Solnik S, Latash ML (2017) Anticipatory postural adjustments and anticipatory synergy adjustments: preparing to a postural perturbation with predictable and unpredictable direction. Exp Brain Res 235:713–730. 10.1007/s00221-016-4835-x 27866261 10.1007/s00221-016-4835-x PMC5316309

[CR40] Riach CL, Hayes KC, Lucy SD (1992) Changes in centre of pressure of ground reaction forces prior to rapid arm movement in normal subjects and patients with cerebellar ataxia. Clin Biomech 7(4):208–214. 10.1016/S0268-0033(92)90003-M 10.1016/S0268-0033(92)90003-M 23915785

[CR41] Robert T, Zatsiorsky VM, Latash ML (2008) Multi-muscle synergies in an unusual postural task: quick shear force production. Exp Brain Res 187:237–253. 10.1007/s00221-008-1299-7 18278488 10.1007/s00221-008-1299-7 PMC2914157

[CR42] Robert T, Bennett BC, Russell SD, Zirker CA, Abel MF (2009) Angular momentum synergies during walking. Exp Brain Res 197:185–197. 10.1007/s00221-009-1904-4 19578841 10.1007/s00221-009-1904-4

[CR43] Schaal S, Sternad D, Osu R, Kawato M (2004) Rhythmic arm movement is not discrete. Nat Neurosci 7(10):1136–1143. 10.1038/nn1322 15452580 10.1038/nn1322

[CR44] Scholz JP, Kelso JS (1990) Intentional switching between patterns of bimanual coordination depends on the intrinsic dynamics of the patterns. J Mot Behav 22(1):98–124. 10.1080/00222895.1990.10735504 15111283 10.1080/00222895.1990.10735504

[CR45] Scholz JP, Schöner G (1999) The uncontrolled manifold concept: identifying control variables for a functional task. Exp Brain Res 126:289–306. 10.1007/s002210050738 10382616 10.1007/s002210050738

[CR46] Scott S (2004) Optimal feedback control and the neural basis of volitional motor control. Nat Rev Neurosci 5(7):532–545. 10.1038/nrn1427 15208695 10.1038/nrn1427

[CR47] Shim JK, Olafsdottir H, Zatsiorsky VM, Latash ML (2005) The emergence and disappearance of multi-digit synergies during force-production tasks. Exp Brain Res 164:260–270. 10.1007/s00221-005-2248-3 15770477 10.1007/s00221-005-2248-3 PMC2826980

[CR48] Shim JK, Park J, Zatsiorsky VM, Latash ML (2006) Adjustments of prehension synergies in response to self-triggered and experimenter-triggered load and torque perturbations. Exp Brain Res 175(4):641–653. 10.1007/s00221-006-0583-7 16804720 10.1007/s00221-006-0583-7 PMC2821078

[CR49] Song J, Shin N, Kim K, Park J (2021) Changes in intersegmental stability during gait in patients with spastic cerebral palsy. Gait Posture 88:264–271. 10.1016/j.gaitpost.2021.06.002 34144330 10.1016/j.gaitpost.2021.06.002

[CR50] Song J, Kim K, Park J (2023) Multi-muscle synergies of postural control in self- and external-triggered force release during simulated archery shooting. J Mot Behav 55(3):289–301. 10.1080/00222895.2023.2187336 36919981 10.1080/00222895.2023.2187336

[CR51] Todorov E, Jordan MI (2002) Optimal feedback control as a theory of motor coordination. Nat Neurosci 5:1226–1235. 10.1038/nn963 12404008 10.1038/nn963

[CR52] Weiler J, Gribble PL, Pruszynski JA (2015) Goal-dependent modulation of the long-latency stretch response at the shoulder, elbow, and wrist. J Neurophysiol 114(6):3242–3254. 10.1152/jn.00702.2015 26445871 10.1152/jn.00702.2015 PMC4686281

[CR53] Worsley KJ, Taylor JE, Tomaiuolo F, Lerch J (2004) Unified univariate and multivariate random field theory. Neuroimage 23:S189–S195. 10.1016/j.neuroimage.2004.07.026 15501088 10.1016/j.neuroimage.2004.07.026

[CR54] Zhou T, Wu Y-H, Bartsch A, Cuadra C, Zatsiorsky VM, Latash ML (2013) Anticipatory synergy adjustments: preparing a quick action in an unknown direction. Exp Brain Res 226:565–573. 10.1007/s00221-013-3469-5 23494385 10.1007/s00221-013-3469-5 PMC3634869

